# Self-Consciousness of Appearance in Chinese Patients With Cleft Lip: Validation of the Chinese Derriford Appearance Scale 59 (DAS 59) Instrument

**DOI:** 10.3389/fped.2021.825997

**Published:** 2022-02-09

**Authors:** Karim A. Sakran, Sixing Song, Huo Li, Peiyue Pan, Nan Chen, Ni Zeng, Ting Chi, Bing Shi, Hanyao Huang, Yan Wang, Caixia Gong

**Affiliations:** ^1^State Key Laboratory of Oral Diseases & National Clinical Research Center for Oral Diseases & Department of Oral Maxillofacial Surgery, West China Hospital of Stomatology, Sichuan University, Chengdu, China; ^2^Department of Pharmacy, Personalized Drug Therapy Key Laboratory of Sichuan Province Sichuan Academy of Medical Sciences & Sichuan Provincial People's Hospital, School of Medicine, University of Electronic Science and Technology of China, Chengdu, China; ^3^Department of Epidemiology and Health Statistics, West China School of Public Health and West China Fourth Hospital, Sichuan University, Chengdu, China

**Keywords:** Derriford Appearance Scale, reliability, quality of life, validation, cleft lip

## Abstract

**Objective:**

To develop a reliable and valid Chinese version of the Derriford Appearance Scale 59 (DAS 59) instrument for assessing the self-consciousness of appearance in Chinese patients with cleft lip.

**Methods:**

The original DAS 59 instrument was translated into Mandarin, back-translated, and culturally adapted among the Chinese population, following the protocol of the original DAS 59. The validation of the Chinese DAS 59 instrument was estimated on 443 adult participants including 213 subjects with a history of cleft lip with/without palate (CL/P, study group) and 230 normal subjects without facial appearance concern (control group). The reliability was estimated by Cronbach's α coefficient and Guttman's split-half coefficient. Content validity was tested using the Spearman correlation coefficient, while discriminant validity was tested by the Mann–Whitney U test.

**Results:**

The overall internal consistency of Chinese DAS 59 was excellent; Cronbach's α was 0.951 (α = 0.965 and 0.959 in the study and control groups, respectively). Further, Guttman's split-half coefficient was excellent in the study group (0.935) and control group (0.901). The validity of content was good with an acceptable correlation between all the items and domains. The construct validity through the discrimination was good with a statistically significant difference in most domains between the two groups. Patients with CL/P had more concern about the general self-consciousness and social self-consciousness of appearance. They also showed a good self-concept score.

**Conclusion:**

The Chinese version of DAS 59 demonstrated acceptable reliability and good construct and discriminant validity. It can be used for the research and assessment of the psychological state and quality of life for Chinese patients with cleft lip as well as other appearance problems and concerns.

## Introduction

Cleft lip with/without palate (CL/P) is a common congenital developmental malformation of the oral and maxillofacial region, accompanied by severe physical defects and various psychological problems (1). The psychological state of CL/P patients can be different from that of normal people (2). Hence, psychological intervention is also a key part of CL/P sequential therapy, which shows a crucial role in the treatment and quality of life (QoL) of patients with CL/P (3).

Physical attractiveness is commonly estimated as a prediction for the individual character and is esteemed independent of other traits. Consequently, physical beauty could affect upon how others see people, with the end goal that alluring people get special treatment during youth and adulthood in most social circumstances. Disfigurement and deformity caused by congenital malformations, diseases, trauma, and burns in addition to their treatments lay apparently various individuals at a social drawback with a danger of heavy trouble and social brokenness (4–6). Therefore, the psychological aspects of deformity should be estimated well, as regular social communication for those with appearance issues is a foundation of unalleviated stress, anxiety, and pain. Moreover, it can be used with surgical interventions to test variations from baseline to post-operation stage, like the benefit inferred from pre- and post-operative photographs (7).

The primary objective of CL/P treatment is to improve appearance and function to get better QoL. To decide if CL/P therapy achieves its proposed target, investigations more often depend on estimating and depicting the professionally reported results, for example, the clinical marks of surgical competence or aesthetics dependent on expert perception (8, 9). Estimating clinician-reported results is critical to set up the clinical adequacy of treatment and guarantee beneficial practice. Nonetheless, relying on clinician-reported results implies significantly less familiarity with the patient-reported results of repair at the end of cleft treatment pathway (10, 11). As of late, there has been a more prominent acknowledgment of the demand concerning the patient's perception in deciding the genuine result of surgery after the treatment of patients with oral cleft (8, 12). However, there is still a lack of comprehensive, valid, and reliable tools that could help in understanding the psychological status of patients with CL/P.

In the past, the investigation regarding this scope was restricted by the lack of a suitable effect measure to estimate individual fulfillment and health-associated QoL. Later on, various psychometric instruments have been developed to evaluate the psychological influence and alteration of manifestation such as the Appearance Schemas Inventory (13), Body Image Avoidance Questionnaire (14), and Body Dysmorphic Disorder Examination (15); however, most of them were criticized for weak content validity, a limited range of applicability, or restricted psychometric development (7). Most of the aforementioned tools did not develop obviously to estimate the range of symptomatology that is applicable to the wide scope of hardships experienced by individuals living with issues of appearance. Thereby, such measures reported lower response to the nature of the dysfunctions and the seriousness of the misery that the involved individuals experience (7).

There is a demand for the establishment and validation of an instrument to estimate patient-reported outcomes in terms of satisfaction, psychosocial prosperity, and health-associated QoL in Chinese patients with cleft lip. Among the patient-reported outcome instruments, the CLEFT-Q is an instrument that was developed internationally for children and young adults with CLP (16, 17). It includes 12 independently functioning scales that evaluate different concerns in terms of appearance, facial function (speech), and health-related QoL. The Derriford Appearance Scale (DAS) was designed by Carr et al. (7) to measure the psychosocial adjustment in patients with appearance issue. The original DAS had shown sustainable reliability and validity and had been utilized in a wide range of populations, including those who had (no) appearance concern in both general and clinical individuals. Two forms of DAS had been developed, one with 24 items, which is mainly composed for daily application in clinical work and another with 59 items, which is intended for research and deep estimation (18, 19). The DAS had also exhibited good psychometric features when translated and approved into different cultural nations (20–23).

Prior to applying any psychometric measure to various cultural group settings, it ought to be translated, approved, and adjusted to regional cultural and social requirements. Thus, the present study was sought to establish a valid and reliable Chinese version of the Derriford Appearance Scale 59 (DAS 59) instrument for assessing the self-consciousness of appearance in Chinese patients with cleft lip.

## Materials and Methods

### Translation of DAS 59 Instrument

The Chinese translation of the DAS 59 instrument was conducted according to the protocol mentioned in the original DAS (7). A native Chinese-speaking surgeon, who was additionally familiar with English, translated the DAS 59 tool into Chinese. A board of Chinese mother-tongue specialists was gathered, which involved five surgeons who lived in an English-native country for more than 1 year. The committee talked about some disputable words utilized in the translation. A backward translation of the Chinese DAS 59 tool into English was conducted by an individual with experience in English linguistics who was not aware with the health-related quality of life (HRQoL) inventories. A specialist board-checked the backward-translated English edition and reconciled it again with the first English one. The last Chinese DAS 59 tool was pretested with a suitable group of patients with/without appearance concerns (40 patients with CL/P and 40 normal subjects) to confirm the readability. After a minor adjustment by the board, the final Chinese DAS 59 tool was developed.

### The Chinese DAS 59 Instrument

Similar to the original DAS 59 (7), the Chinese DAS 59 is formulated as a sequence of 59 statements and questions with reply categories in a Likert format from 1 to 5 to assess the frequency of symptoms (1, almost never.... 5, almost always) and levels of distress (1, not at all distressed.... 5, extremely distressed). It is intended for use in subjects aged ≥16 years. An introductory part gathers the features of appearance aspect to which the respondent raises most sensitivity. This is meant as the respondent “feature” in the body of the instrument. The instrument also detects any other aspects of appearance to which the person might also have distresses. A total of 57 items assess the range of psychological discomfort and dysfunction, and 2 items assess physical distress and physical dysfunction. The instrument was developed to be utilized by clinical and scientific experts from relevant fields of plastic surgery, dermatology, clinical psychology, and psychiatry.

Simple and concise guidelines are given on how to accomplish the DAS 59, which is composed as a self-report instrument to be finished without others' intervention. The DAS 59 creates six aspects of psychological discomfort and dysfunction (total score and five domain scores) in addition to an aspect of physical discomfort and dysfunction. The five domains are (1) General self-consciousness of appearance (GSC); (2) Social self-consciousness of appearance (SSC); (3) Sexual and body self-consciousness of appearance (SBSC); (4) Negative self-concept (NSC); and (5) Facial self-consciousness of appearance (FSC). The greater the scale, the higher the severity of discomfort and dysfunction. Total scale and domain scores are achieved by adding the scores of independent items as indicated by the instructions presented in a guide that supplies the original DAS 59 (24).

### Sample Size Calculation

The required sample for internal consistency of the Cronbach's alpha was computed by utilizing Bonnett's formula (25) with an alpha of 0.05 and a power of 90%, 133 participants would be required. Ethical approval was obtained from the ethical committee of West China Hospital of Stomatology, Sichuan University (No. WCHSIRB-D-2016-084R1), and the guidelines from the Declaration of Helsinki were followed.

### Study Participants

A total of 218 adult patients with a history of CL/P who visited the center of cleft lip and palate, West China Hospital of Stomatology, Sichuan University from March 2018 to October 2019 were enrolled as a study group. A control group of 230 normal adults who did not have appearance-related concerns and were free of any previous history of cosmetic-intended surgical interventions were conveniently enrolled from outpatient clinics in the same area of the study. Therefore, the Chinese DAS 59 instrument was delivered to a total of 448 participants after being instructed about the objective of the project and singed an informed consent form. The participants completed the Chinese DAS 59 independently within 15 min under the guidance of an assistant who did not interfere with the privacy of the subjects. Finally, there were 443 participants who completed the instrument, accounting for 98.9% of delivered reports; 213 in the study group (129 males and 84 females) aged between 16 and 64 years (mean age = 22.53 ± 7.87 years) and 230 in the control group (96 males and 134 females) aged between 18 and 65 years (mean age = 28.47 ± 10.42 years).

### Statistical Analysis

IBM SPSS version 25 (IBM Corp., Armonk, NY, USA) was utilized to do the analysis. The reliability of the DAS 59 instrument was tested by the Cronbach's alpha coefficient and Guttman's split-half coefficient. It was regarded sustainable when the alpha was ≥0.70 and satisfactory for those ≥0.60 (26). The validity of content was tested by estimating the correlations between the items and also the domains using the Spearman correlation coefficient. Discriminant validity was evaluated by assessing the variations between the two groups using the Mann–Whitney U test. The reports were not considered if there are two or more missing values. If a report was presented with one missed value, such value was compensated by the mean score of the relevant item (26).

## Results

### Reliability

The Chinese DAS 59 instrument revealed excellent reliability and internal consistency as demonstrated by a total excellent Cronbach's alpha coefficient (0.961, Table 1) and Guttman's split-half coefficient (0.931, Table 2). The five main domains of DAS 59: GSC, SSC, SBSC, NSC, and FSC, have revealed good reliability as revealed by the total Cronbach's alpha values of 0.923, 0.928, 0.774, 0.885, and 0.723, respectively.

**Table 1:** Reliability and internal consistency of Chinese DAS 59 using Cronbach's alpha.

**Domain**	**Item no**.	**Study group**	**Control group**	**Overall**
General self-consciousness of appearance	17	0.942	0.891	0.923
Social self-consciousness of appearance	20	0.940	0.909	0.928
Sexual and bodily self-consciousness of appearance	9	0.775	0.780	0.774
Negative self-concept	5	0.876	0.846	0.885
Facial self-consciousness of appearance	4	0.750	0.686	0.723
Physics	2	0.716	0.916	0.798
No factor	2	0.507	0.457	0.479
Total	59	0.965	0.959	0.961

**Table 2:** Reliability and internal consistency of Chinese DAS 59 using Guttman's split-half coefficient.

**Domain**	**Item no**.	**Study group**	**Control group**	**Overall**
GSC	9	0.909	0.819	0.879
SSC	10	0.898	0.835	0.874
SBSC	5	0.731	0.660	0.708
NSC	3	0.842	0.789	0.860
FSC	2	0.744	0.678	0.711
Physics	1	0.716	0.916	0.798
No factor	1	0.507	0.457	0.479
Total	31	0.935	0.901	0.913

The Guttman's split-half coefficient also confirmed the good reliability of these five main domains as shown in Table 2.

### Validity

The face validity of Chinese DAS 59 was good as confirmed by the expert committee and was further confirmed by a review of literature. The content validity was also good as revealed by the significant correlation between almost all the items and domains (Table 3). Discriminant validity was proven by the significant variations between the study and control groups (Table 4).

**Table 3:** Spearman's correlation coefficient between domains of Chinese DAS 59.

**Domain**	**GSC**	**SSC**	**SBSC**	**NSC**	**FSC**	**Physics**	**No factor**
GSC	1.000	0.869^**^	0.784^**^	0.127^**^	0.635^**^	0.616^**^	0.632^**^
SSC	0.869^**^	1.000	0.803^**^	0.077	0.710^**^	0.637^**^	0.620^**^
SBSC	0.784^**^	0.803^**^	1.000	0.047	0.781^**^	0.637^**^	0.587^**^
NSC	0.127^**^	0.077	0.047	1.000	0.023	0.069	0.068
FSC	0.635^**^	0.710^**^	0.781^**^	0.023	1.000	0.566^**^	0.502^**^
Physics	0.616^**^	0.637^**^	0.637^**^	0.069	0.566^**^	1.000	0.457^**^
No factor	0.632^**^	0.620^**^	0.587^**^	0.068	0.502^**^	0.457^**^	1.000

** *Correlation is significant at the 0.01 level (two-tailed)*.

**Table 4:** Discriminant validity of Chinese DAS 59.

**Domain**	***P*-value**
GSC	0.684
SSC	0.025
SBSC	0.002
NSC	0.000
FSC	0.000
Total score	0.638

*Statistical significance at 0.05 level*.

### Self-Consciousness of Appearance in Patients With Cleft Lip

Tables 5, 6 show the scores of DAS 59 responses for patients with cleft lip. Overall, patients with cleft lip have shown high concerns regarding their appearance, particularly in terms of the general self-consciousness of appearance and social self-consciousness of appearance. On the other hand, they had shown a good degree of positive self-concept.

**Table 5:** Total and domain scores of DAS 59 scale in patients with cleft lip.

**Domain**	**Min**	**Q1**	**Q2**	**Q3**	**Max**	**Mean (SD)**
GSC	17	30	41	52	83	41.8 (14.7)
SSC	20	27	39	54	85	41.1 (16)
SBSC	9	13.5	19	24	41	19.5 (7.4)
NSC	5	13	16	20	25	16.1 (5)
FSC	4	4.5	7	10	18	7.8 (3.4)
Physics	2	2	3	5	10	3.7 (1.9)
No factor	2	3	4	6	10	4.3 (1.8)
Total score	59	103	130	164.5	244	134.3 (40.8)

**Table 6:** Score of domain's items of DAS 59 scale in patients with cleft lip.

**Domain's items**	**Min**	**Q1**	**Q2**	**Q3**	**Max**	**Mean (SD)**
**General self-consciousness of appearance**						
1. Self-consciousness of “feature”	1	3	3	4	5	3.1 (1.1)
8. Taking a special interest in others' “features”	1	1.5	3	3	5	2.5 (1.1)
10. Avoiding photography	1	2	3	4	5	2.8 (1.2)
12. Being hurt by others' comments	1	2	3	4	5	3 (1.3)
15. Raising subject of the “feature” in conversation before others do	0	1	2	3	5	2.3 (1.1)
17. Being irritable at home	1	1	2	3	5	2.3 (1.1)
27. Feel unattractive	1	1	2	3	5	2.4 (1.2)
28. Feel unlovable	1	1	2	3	5	2 (1.1)
30. Feel embarrassed	1	1	2	3	5	2.3 (1.2)
31. Feel inferior	1	1	2	3	5	2.5 (1.3)
34. Distress when others stare	1	1	2	3	5	2.3 (1.2)
35. Distress when others make remarks	1	1	3	3	5	2.6 (1.3)
36. Distress when others ask about the “feature”	1	1	3	3	5	2.6 (1.3)
38. Distress when seen in a particular view	1	1	2	3	5	2.5 (1.3)
41. Distress when “feature” seen in a mirror/window	1	1	2	3	5	2.3 (1.3)
42. Distress when meeting strangers	1	1	2	3	5	2.2 (1.2)
58. How hurt do you feel?	1	1	2	3	5	2.3 (1.1)
**Social self-consciousness of appearance**						
2. Avoiding children in the street	1	1	2	3	5	2 (1.2)
3. Difficulty making friends	1	1	2	3	5	2.3 (1.3)
5. Avoiding school/college/work	1	1	1	3	5	2 (1.2)
6. Avoiding pubs/restaurants	1	1	1	3	5	1.8 (1.1)
7. Avoiding parties/discos	1	1	2	3	5	2.2 (1.3)
13. Avoiding department stores	1	1	1	3	5	1.9 (1.1)
14. Avoid leaving the house	1	1	2	3	5	2.3 (1.2)
16. Closing into a shell	1	1	2	3	5	2.1 (1.2)
18. Being misjudged	1	1	3	3	5	2.5 (1.2)
19. Previous avoidance of school/college/work	0	1	1	3	5	1.9 (1.2)
20. Feeling an embarrassment to friends	1	1	2	3	5	2.1 (1.1)
21. Feeling a freak	1	1	2	3	5	2 (1.2)
22. Worrying about sanity	1	1	1	2	5	1.7 (1.1)
29. Feel isolated	1	1	2	3	5	2.4 (1.3)
32. Feel rejected	1	1	2	3	5	2.3 (1.2)
33. Feel useless	1	1	2	3	5	1.9 (1.1)
39. Distress when going to school/college/work	1	1	2	3	5	2.2 (1.2)
40. Distress when on public transport	1	1	1	3	5	1.9 (1.1)
47. Distress when not being able to go to social events	1	1	2	3	5	2 (1.2)
50. Distress when not being able to go to pubs/restaurants	1	1	1	3	5	1.8 (1.1)
**Sexual and bodily self-consciousness of appearance**						
4. Avoiding undressing in front of partner	1	1.5	3	3.5	5	2.7 (1.3)
9. Avoiding communal changing rooms	1	1	3	3	5	2.4 (1.2)
23. Adverse effect on sex life	1	1	2	3	5	2 (1.2)
24. Adverse effect on marriage	1	1	2	3	5	2.2 (1.3)
37. Distress when going to the beach	1	1	2	3	5	2.1 (1.2)
43. Distress from being unable to wear favorite clothes	1	1	2	3	5	2.3 (1.3)
45. Distress from being unable to go swimming	0	1	1	3	5	1.8 (1.1)
46. Distress from being unable to play games	1	1	2	3	5	2 (1.1)
49. Distress from being unable to look in the mirror	1	1	1	3	5	1.9 (1.2)
**Negative self-concept**						
52. How confident do you feel?	1	2	3	4	5	3.1 (1.2)
54. How secure do you feel?	1	2	3	4	5	3.1 (1.2)
55. How cheerful do you feel?	1	3	3	4	5	3.3 (1.2)
56. How normal do you feel?	1	3	3	4	5	3.5 (1.2)
57. How masculine/feminine do you feel?	1	3	3	4	5	3.2 (1.3)
**Facial self-consciousness of appearance**						
11. Avoid getting the hair wet	1	1	2	3	5	2.2 (1.2)
44. Distress from being unable to change hairstyle	1	1	2	3	5	2.1 (1.2)
48. Distress from being unable to answer the front door	1	1	1	3	5	1.8 (1)
51. Distress from being unable to go out in windy weather	1	1	1	3	5	1.8 (1.1)
**Physics**						
25. Cause physical pain	1	1	1	2	5	1.7 (1)
26. There are physical limitations	1	1	1	3	5	2 (1.2)
**No specific factor**						
53. How irritable do you feel?	1	1.5	3	3	5	2.5 (1.2)
59. How hostile do you feel?	1	1	1	3	5	1.8 (1.1)

## Discussion

More recently, the QoL has turned into a valuable parameter in evaluating the viability of clinical interventions. An evaluation of the results relevant to QoL is critical in aesthetic surgery, like CL/P treatment, since patient satisfaction is the transcendent element by which achievement is characterized. Until a later time, it was hard to validate these disputes in an objective design. The previous period, nonetheless, has realized a blast of concern in QoL evaluation devices as a substitute tool for generally profiting from health interventions (27). A progression of tools relevant to health-associated QoL are presently accessible; a large portion of them are instruments with non-specific expressions, not explicitly made for subjects going through aesthetic surgery, and they might misjudge specific impacts of body modifications coming from these interventions.

In the modern time, investigations have started to consider the construction of self-perception and its connection to corrective clinical treatments (28). Experimental proof reflected by some developing studies proposes that aesthetic patients reveal self-perception disappointment at baseline and improvements in self-perception after the operation. Estimating patient-centered outcomes have become progressively critical in aesthetic and reconstructive surgical procedures.

The DAS is a tool developed to precisely and reliably assess the variety of the QoL after cosmetic and reconstructive surgical interventions. The DAS was particularly intended to assess the psychosocial change in individuals who have reported cosmetic issues. This instrument has shown sustainable reliability and validity and had been used in a wide range of people, including those with/without appearance concerns.

The present study describes the translation and validation of the Chinese form of the DAS 59 instrument and its application to patients with CL/P. The translation procedure was performed, following the protocol mentioned in the original DAS. The Chinese DAS enrolled a clinical population represented by cleft lip patients and normal population without appearance concerns. The total internal consistency was excellent (α = 0.96) and was quite close to the alpha value reported in the original DAS 59 (7). All the items have demonstrated good correlation, revealing a good homogeneity. Further, domains also exhibited sustainable internal consistency and correlation.

The construct validity of the Chinese DAS 59 was tested through discrimination between the involved groups. Discriminant validity was confirmed by significant variations among the CL/P subjects and normal population. In this context, the social self-consciousness of appearance, sexual and bodily self-consciousness of appearance, negative self-concept, and facial self-consciousness of appearance have good discriminating validity between two groups. Meanwhile, it can be reflected in the patients with CL/P that maxillofacial defects have obvious influence on social life and other aspects of clinical patients. However, in terms of the general self-consciousness of appearance, and physical influence, there was no obvious variation between the two groups. These findings could be referred to some various demographic characteristics between the two groups such as occupation and education level.

Patients with cleft lip showed almost high concerns about their appearance and associated psychosocial aspects as revealed by their self-reported scores in DAS 59 responses. Their concerns were more prominent in the aspect of general self-consciousness of appearance (41.8 ± 14.7), particularly in the items of self-consciousness of “feature,” taking a special interest in others' “features,” avoiding photography, being hurt by others' comments, distress when others make remarks, and distress when others ask about the ‘feature'. Moreover, they also gave an apparent concern about their social self-consciousness of appearance (41.1 ± 16), particularly in the items of being misjudged, difficulty making friends, avoiding parties/discos, avoid leaving the house, feel isolated, feel rejected, and distress when going to school/college/work. On the other hand, patients with cleft lip still showed an apparent degree of positive self-concept as shown in their responses of items under the negative self-concept domain.

More and less, the current study further confirms that the DAS 59 could be a valuable tool for assessing the viability of cosmetic and reconstructive intervention and going further to analyze the motivations underlying the demand of appearance-related interventions. Although the present study included an acceptable sample size, it is still not enough for a more comprehensive analysis of the DAS 59 instrument. Hence, the present cohort did not investigate the factorial construction of the Chinese DAS 59. In addition, the present study had only estimated the validation of DAS 59 in a limited range of clinical samples (CL/P). Therefore, a multicenter research that includes a larger sample size with a wide diversity of clinical population will be necessary to get a more robust result and thorough exploring of the different aspects underlying the DAS 59.

## Conclusion

A reliable and valid Chinese form of DAS 59 was established to assess the psychological impact in individuals with appearance concerns. Patients with cleft lip have shown a high concern regarding their appearance, especially to the general and social self-consciousness of appearance. Thereby, the Chinese DAS 59 instrument could be useful in assessing and understanding the psychological and psychosocial distress of Chinese patients with cleft lip. The present Chinese DAS 59 instrument could be approved more by further validation and improvement study.

## Data Availability Statement

The original contributions presented in the study are included in the article/supplementary material, further inquiries can be directed to the corresponding author/s.

## Ethics Statement

The studies involving human participants were reviewed and approved by the Institutional Ethical Review Board of West China Hospital of Stomatology, Sichuan University (approval no. WCHSIRB-D-2016-084R1). Written informed consent to participate in this study was provided by the participants' legal guardian/next of kin.

## Author Contributions

KS, SS, HL, PP, NC, and TC contributed to the collection of data. KS, SS, HL, NZ, and TC analyzed the data. KS, SS, HL, HH, YW, and CG contributed to writing and revising the article. BS, HH, YW, and CG supervised the research. All authors contributed to the article and approved the submitted version.

## Funding

This work was supported by the Research and Develop Program, West China Hospital of Stomatology Sichuan University grant to HH (RD-02-202107) and the National Natural Science Foundation of China grant to BS (81974147).

## Conflict of Interest

The authors declare that the research was conducted in the absence of any commercial or financial relationships that could be construed as a potential conflict of interest.

## Publisher's Note

All claims expressed in this article are solely those of the authors and do not necessarily represent those of their affiliated organizations, or those of the publisher, the editors and the reviewers. Any product that may be evaluated in this article, or claim that may be made by its manufacturer, is not guaranteed or endorsed by the publisher.
